# Impact of Recombinant VSV-HIV Prime, DNA-Boost Vaccine Candidates on Immunogenicity and Viremia on SHIV-Infected Rhesus Macaques

**DOI:** 10.3390/vaccines12040369

**Published:** 2024-03-29

**Authors:** Alice Berger, Jannie Pedersen, Monika M. Kowatsch, Florine Scholte, Marc-Alexandre Lafrance, Hiva Azizi, Yue Li, Alejandro Gomez, Matthew Wade, Hugues Fausther-Bovendo, Marc-Antoine de La Vega, Joseph Jelinski, George Babuadze, Marie-Edith Nepveu-Traversy, Claude Lamarre, Trina Racine, Chil-Yong Kang, Bruno Gaillet, Alain Garnier, Rénald Gilbert, Amine Kamen, Xiao-Jian Yao, Keith R. Fowke, Eric Arts, Gary Kobinger

**Affiliations:** 1 Département de Microbiologie-Infectiologie et Immunologie, Faculté de Médecine, Unversité Laval, Quebec, QC G1V 0A6, Canada; ecila.berger@gmail.com (A.B.); jannie.pedersen3@rsyd.dk (J.P.); kyj7@cdc.gov (F.S.); marc-alexandre.lafrance.1@ulaval.ca (M.-A.L.); hiva.azizi@nrc-cnrc.gc.ca (H.A.); alemgomez@gmail.com (A.G.); matthew.wade6@icloud.com (M.W.); hffausth@utmb.edu (H.F.-B.); madelave@utmb.edu (M.-A.d.L.V.); gebabuad@utmb.edu (G.B.); claude.lamar@cervo.ulaval.ca (C.L.); 2 Department of Medical Microbiology and Infectious Diseases, University of Manitoba, Winnipeg, MB R3T 2N2, Canada; umkowats@myumanitoba.ca (M.M.K.); keith.fowke@umanitoba.ca (K.R.F.); 3 Department of Microbiology and Immunology, Schulich School of Medicine and Dentistry, University of Western Ontario, London, ON N6A 3K7, Canada; yli3685@uwo.ca (Y.L.); cykang@uwo.ca (C.-Y.K.); ericjarts@gmail.com (E.A.); 4 Galveston National Laboratory, Department of Microbiology and Immunology, University of Texas Medical Branch, Galveston, TX 77555, USA; jojelins@utmb.edu; 5 Global Urgent and Advanced Research and Development, Batiscan, QC G0X 1A0, Canada; mntraversy@guardrx.org; 6 Axe des Maladies Infectieuses et Immunitaires, Centre de Recherche du Centre Hospitalier Universitaire de Québec-Université Laval, Quebec, QC G1E 6W2, Canada; trina.racine@usask.ca (T.R.); xiao-jian.yao@umanitoba.ca (X.-J.Y.); 7 Department of Chemical Engineering, Faculty of Science and Engineering, Laval University, Quebec, QC G1V 0A6, Canada; bruno.gaillet@gch.ulaval.ca (B.G.); alain.garnier@gch.ulaval.ca (A.G.); 8 Department of Production Platforms and Analytics, Human Health Therapeutics Research Center, National Research Council, Montreal, QC H4P 2R2, Canada; renald.gilbert@cnrc-nrc.gc.ca; 9 Department of Bioengineering, McGill University, Montreal, QC H3A 0G4, Canada; amine.kamen@mcgill.ca

**Keywords:** VSV vaccine, DNA vaccine, HIV, SHIV, rhesus macaque

## Abstract

Currently, no effective vaccine to prevent human immunodeficiency virus (HIV) infection is available, and various platforms are being examined. The vesicular stomatitis virus (VSV) vaccine vehicle can induce robust humoral and cell-mediated immune responses, making it a suitable candidate for the development of an HIV vaccine. Here, we analyze the protective immunological impacts of recombinant VSV vaccine vectors that express chimeric HIV Envelope proteins (Env) in rhesus macaques. To improve the immunogenicity of these VSV-HIV Env vaccine candidates, we generated chimeric Envs containing the transmembrane and cytoplasmic tail of the simian immunodeficiency virus (SIV), which increases surface Env on the particle. Additionally, the Ebola virus glycoprotein was added to the VSV-HIV vaccine particles to divert tropism from CD4 T cells and enhance their replications both in vitro and in vivo. Animals were boosted with DNA constructs that encoded matching antigens. Vaccinated animals developed non-neutralizing antibody responses against both the HIV Env and the Ebola virus glycoprotein (EBOV GP) as well as systemic memory T-cell activation. However, these responses were not associated with observable protection against simian-HIV (SHIV) infection following repeated high-dose intra-rectal SHIV SF162p3 challenges.

## 1. Introduction

Despite decades of intensive research, a protective vaccine against the human immunodeficiency virus (HIV) remains an elusive goal [1] Various vaccine platforms have been tested in different animal models, but efficacy in non-human primate studies and human clinical trials remains low. To date, only the RV144 vaccine trial showed a modest (31%) protection in humans at the end of the 3-year follow-up [2,3], although post hoc analyses showed an estimated cumulative vaccine efficacy of 60% at 12 months following initial vaccinations [4]. Several pre-clinical studies in rhesus macaques using various vaccine platforms for an optimized response have been completed and, although some have reported varying levels of protection (15–71%), no vaccination strategy has been proven optimal thus far [5,6,7,8,9,10,11,12,13,14,15]. In most of these studies, protective efficacy was observed when a combination of different platforms was used, either simultaneously or as a prime-boost strategy. However, no consensus on correlates of protection exists to date [9,13,14,15]. The humoral response to HIV vaccine candidates has been the most thoroughly studied with a focus on broadly neutralizing antibodies (bnAb). Pre-clinical studies have shown that the passive infusion of bnAb can prevent infection, and high levels of bnAb have been reported to play an important role in protection. In the RV144 trial, however, non-neutralizing V1V2-binding antibodies were the primary correlate of protection, followed by HIV-specific CD4 T cells [13,16,17,18].

Vesicular stomatitis virus (VSV)-based vaccines have been widely explored for a broad range of viral targets and have been evaluated in several clinical trials. For example, a recombinant VSV (rVSV) Ebola virus vaccine demonstrated protection in a phase III trial, received regulatory approval in 2019, and has been frequently utilized in Ebola virus outbreaks [19,20,21,22]. There are several advantages of using the VSV vaccine platform, including its ability to induce robust humoral and cell-mediated immune responses [23]. The rVSV-HIV particles can display more Env spikes on their surfaces than genuine HIV-1 virions or virus-like particles (VLPs). This is seen especially when replacing the HIV Env membrane-proximal external regions (MPER) and transmembrane (TM) domains with those of VSV [24]. A recent study examined the expression and immunogenicity in constructs with SIV MPER of TM and reported a 200-fold higher increase in gp140-binding antibodies in mice immunized with rVSV-HIVEnv-SIV-TMCT as compared to HIV-1 MPER-TM [25]. The high-Env density on the surface of the rVSV viral particle and the ability to display the glycoproteins in their natural trimeric form are some of the elements believed to enhance immunogenicity, making these rVSV vectors interesting candidates for further testing [24].

Early studies using an attenuated VSV vector expressing HIV Gag and Env proteins in rhesus macaques showed promising results, underlining the potentially important role of this vector [26,27,28]. *Rose* et al. reported viremic control and saw correlation with both humoral and cellular responses [28]. A subsequent study examining rVSV-HIV vaccines and different administrations pointed to CTL as a possible correlate of protection [27]. Parks et al. reported 67% protection against infection using an rVSVΔG-HIV Env vaccine in a rhesus macaque model and saw high pre-challenge antibodies but no clear protection correlates [26]. However, this latter vaccine candidate was difficult to propagate in vitro, hampering the reproducibility of these results. Further developments on this design used a dual glycoprotein (GP) expression system in rVSV. This concept has previously been tested with HIV Env, in which a new rVSVΔG-HIV was engineered to produce the full-length GP from the lymphocytic choriomeningitis virus (LCMV) in addition to the HIV Env, demonstrating the possibility for dual-GP expressing VSV-based vectors and solving the in vitro propagation obstacle [29]. Precedence for dual GP expression with ebolavirus (EBOV) GP was seen in a VSV-system co-expressing the influenza hemagglutinin (HA) GP, which provided 100% protection against both pathogens in a mouse model [30]. This construct demonstrated the possibility of building on the already clinically evaluated/FDA-approved rVSV-EBOV to create an rVSV dual GP expression system with both HIV and EBOV proteins.

In this study, we evaluated the protective capacity of two HIV-Env sequences as antigens in an HIV vaccination, one from a Clade A source and one from a Clade B source. These chimeric HIV Env with modified cytoplasmatic tails and TM domains were paired with EBOV GP immunogens in a single construct (VSVΔG-HIV Env-EBOV). These constructs were used in a heterologous rVSV prime/DNA boost strategy. Non-human primates were then challenged with repeated high-dose intrarectal SHIV-SF162p3 challenges to determine the efficacy of these vaccines. The results from this study were gathered with the intent to inform future rVSV-HIV vaccine design and were successfully used to elaborate a promising vaccine strategy described in *Jelinski, Kowatsch, and Lafrance* et al. [31].

## 2. Materials and Methods

### 2.1. Vaccine Candidates

The design, construction, and validation of the VSV-HIV vectors have been described elsewhere [32]. In short, two previously published HIV Env sequences, envA74_N425K_ (Clade A) and envNL4.3 (Clade B), were modified to contain the transmembrane domain and cytoplasmic tail of SIV (SIVtmCT) using standard cloning techniques [33,34]. The A74 and NL4.3 Env sequences share 79.8% and 84.4% amino acid sequence identity, respectively, with the SHIV SF162p3 Env sequence. The chimeric Env sequences were cloned into a modified VSVΔG vector, along with an EBOV GP variant (B6) displaying increased tropism for human cells, using similar methodology, and were rescued using Vero E6 and HEK293T cells as previously described [35,36]. The resulting vaccines, VSV-B6-A74env/SIVtm and VSV-B6-NL4.3env/SIVtm, expressed envA74_N425K_ and envNL4.3, respectively. The empty vector, denoted as VSV-B6 stock virus, was grown on Vero E6 cells and purified using a standard sucrose cushion before storage at −80 °C. In addition, the chimeric HIV Env and HIV Rev were cloned into pIDV-II using standard cloning techniques [37].

### 2.2. Animals, Vaccinations, and Challenges

Thirty Chinese-origin female rhesus macaques (*Macaca mulatta*) under 10 kg were housed at the Université Laval animal facility and maintained according to standards outlined by the Canadian Council on Animal Care. Prior to study initiation, ethical protocols were approved by the Université Laval animal protection committee (Protocol number: 2017098-1). The animals were housed in two large enclosures, with 10 animals in the first and 20 in the second, enabling social interaction and group dynamics. The minimal size of each enclosure followed the national guidelines. They had enriched food, a water pool, music, and toys to play with, along with movie entertainment. The food was optimized for the conditions of the animals and served twice daily.

Animal health was monitored by the animal care staff several times a day via visual control; the staff looked for clinical criteria of sickness (signs of dehydration, diarrhea, psychomotor slowing). The animals were weighed weekly and, if needed, blood hematology tests were performed with a VetScan HM5 analyser (Abaxis, Union City, CA, USA). All manipulations were completed under anesthesia with ketamine, and only trained animal care staff carried out these procedures to minimize the suffering of the animals, in accordance with national guidelines.

The randomization of animals into groups was performed by the animal care staff using standard procedures within the facility. Prime immunizations were performed by intra-muscular administration in the quadriceps with 1 × 10^8^ Median Tissue Culture Infectious Dose (TCID50) VSV-B6-A74env/SIVtm (group A74, n = 10) or 2 × 10^8^ TCID50 VSV-B6-NL4.3env/SIVtm (group NL4.3, n = 10). The control group was vaccinated intramuscularly with 1 × 10^8^ TCID_50_ VSV-B6 (n = 10). After 9 weeks, each experimental group was boosted by intradermal vaccination with 1 mg of pIDVII_HIVenvA74N425K.SIVtm (group A74) or pIDV-II_HIVenvNL4.3. SIVtm (group NL4.3). In addition, both groups received 0.4 mg of pIDV-II_rev. The co-expression of HIV Rev was previously shown to result in the increased expression of the chimeric Env protein [37]. Both DNA HIV-vaccine candidates were administered using an intradermal oscillating needle array injection device (IONAID) [38]. The control group was boosted as follows: five animals were boosted with 1 mg of an EBOV GP-DNA vaccine candidate (INO-4212, Inovio, San Diego, CA, USA) through intradermal injection with electroporation, and five animals were mock-boosted with 1 mL of PBS using the IONAID device.

**Figure 1:**
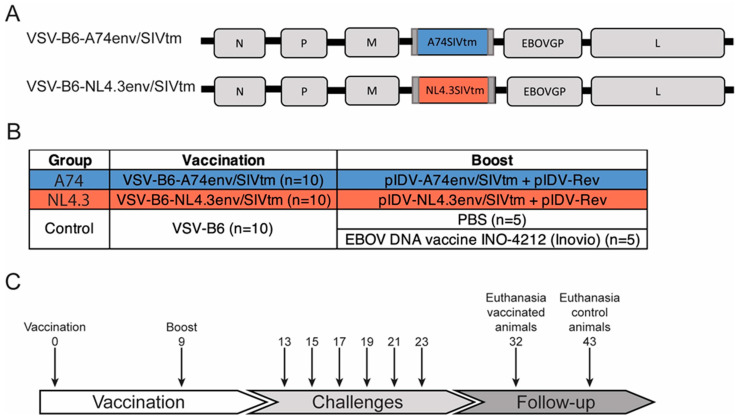
**Vaccine vectors and timelines.** (**A**) Schematic representation of the chimeric VSV vaccine vectors for the experimental groups. (**B**) Experimental and control groups with corresponding vaccination regimes and (**C**) timeline for the study including vaccination schedule, challenges, and follow-up. N: Nucleoprotein, P: Phosphoprotein, M: Matrix protein, L: Polymerase, B6: EBOV GP.

All animals were challenged intrarectally 30 days after the boost with 1 × 10^4^ TCID50 of the HIV-1 clade-B Env virus SHIV SF162p3. The animals were infected every two weeks for a total of six challenges. After the final challenge, the animals were monitored for 9 weeks (group A74 and NL4.3) or 20 weeks (control group) before euthanasia (Figure 1).

### 2.3. ELISA

Binding antibodies against the HIV-1 gp120 M-consensus recombinant protein (M-CONS-S D11gp120), HIV-1 gp140 recombinant protein (B.6240 gp140C) (both from the NIH AIDS Reagent Program) and the recombinant Zaire EBOV GP protein (Cedarlane, Burlington, ON, Canada), were quantified by ELISA. Briefly, Costar Assay 96-well, half area, high-binding, polystyrene plates (Corning, Tewksbury, MA, USA) were coated with 30 ng protein diluted in PBS and incubated overnight at 4 °C. Blocking was completed with Milk Diluent (SeraCare, Milford, MA, USA) for 30 min at room temperature followed by 30 min at 37 °C. Sera were added in 1:200 or 1:400 dilutions in Milk Diluent in triplicate and incubated at room temperature for 2 h. Following washes with PBS + 0.1% Tween-20, goat polyclonal anti-human IgG-HRP (VWR 95058-720) diluted in blocking buffer (1:2000) was added and incubated for 1 h at room temperature. A final wash was performed before detection mix (SeraCare Milford, MA, USA) according to the manufacturer’s instructions and incubated for 30 min at 37 °C before reading the plate using a multi-plate reader (Synergy, BioTek, Shoreline, WA, USA) at a 405 nm wavelength.

### 2.4. Virus Neutralization Assay

The neutralization against Env pseudoviruses (Q23ENV17 (clade A) and pCAGGS SF162 gp160 (clade B)) (NIH AIDS Reagents Program Cat# 10463, 10455) was measured with a luciferase-based assay in TZM-bl cells as previously described [39]. Briefly, three-fold serial dilutions of sera were added in duplicate to TZM-bl cells and incubated for 1 h at 37 °C. 100 TCID50 of virus in DMEM containing 11 μg/mL DEAE-dextran (Sigma-Aldrich, Carlsbad, CA, USA) were added to each well. After a 48-h incubation at 37 °C, the assay medium was removed from each well, and 10 μL of lysis buffer and 60 μL Galacto-Star luciferase reagents (ThermoFisher Scientific, Waltham, MA, USA) were added, and luminescence was measured. The assay controls included TZM-bl cells alone (cell control) and TZM-bl cells with virus (virus control). The IC50 titer was calculated as the antibody dilution that caused a 50% reduction in relative luminescence units (RLUs) compared to the virus control after the subtraction of cell control RLUs.

### 2.5. Plasma Viral Load and Viral Rectal Shedding

During the study, the viral load in plasma and mucosal membranes (rectal swabs) was evaluated by RT-qPCR. To determine the plasma viral load, 1 mL of plasma was centrifugated for 90 min at 20,000× *g* at 4 °C. The pellet was resuspended in a lysis buffer containing 300 µL RLT buffer (RNeasy Mini Kit, Qiagen, Toronto, ON, Canada), 3 µL β-mercaptoethanol, and 16 µL proteinase K solution (ThermoFisher Scientific, Burlington, ON, Canada) and was incubated at 56 °C for 60 min in a water bath. Then, the RNA was purified using spin columns as per the RNeasy Mini kit protocol. The RNA was eluted in 50 µL of Rnase-free water supplemented with 1 mM dithiothreitol (ThermoFisher Scientific, Burlington, ON, Canada) and 1 U/mL RNAse Inhibitor (Qiagen, Toronto, ON Canada). Gag-specific forward (5′-GTCTGCGTCATCTGGTGCAT-3′) and reverse (5′-CACTAGCTGTCTCTGCACTATGT-GTTT-3′) primers and a 6-carboxyfluorescein (FAM)-labeled minor groove binder (MGB) probe (5′-6FAM-CTTCCTCAGTGTGTTTCA-MGB-3′) were obtained from Integrated DNA Technologies (IDT, Coralville, IA, USA). For viral load quantification, a synthesized SIV-gag RNA standard (IDT, Coralville, IA, USA) was used. Reverse transcription was performed using a Sensiscript RT kit (Qiagen, Toronto, ON, Canada), followed by qPCR using a LightCycler 480 Probes qPCR Master Mix (Roche, Laval, QC, Canada). The reaction was run on a LightCycler 480. The assay sensitivity was >165 copies/mL, and the positivity cut-off was set at >300 copies/mL.

To assess viral rectal shedding, tubes containing rectal swabs in Dulbecco’s Modified Eagle Medium (DMEM) were thawed and vortexed for 15 s. The swabs were discarded, and the remaining media were centrifuged for 10 min at 1500× *g* at 4 °C to pellet cells and debris. The supernatant was collected, and 400 µL were centrifuged for 90 min at 20,000× *g* at 4 °C to pellet the virus. The pellets were resuspended in AVL lysis buffer containing carrier RNA, and the RNA was purified using spin columns following the QIAamp Viral RNA Mini protocol (Qiagen, Toronto, ON, Canada). RT and qPCR were performed as described above. The assay sensitivity was set at 666 copies/swab.

### 2.6. T-Cell Responses

Peripheral blood mononuclear cells (PBMCs) were isolated from whole blood collected in K2 EDTA Vacutainer tubes (BD Biosciences, Franklin Lakes, NJ, USA) using Ficoll-Paque density gradient (GE Healthcare, Boston, MA, USA). The cells were frozen in fetal bovine serum (FBS) (Wisent bioproducts, St-Bruno, QC, Canada) with 10% DMSO and stored in liquid nitrogen until use. PBMCs were thawed and then rested for 4 h. HIV-specific T-cell cytokine production and proliferation were evaluated after a 12-h or a 7-day stimulation, respectively. For both assays, PBMCs were stimulated with 0.5 µg/mL HIV Env peptide pool (Group A74; HIV Subtype A1, Group NL4.3; Subtype B (NIH AIDS Research Program, Bethesda, MD, USA)) in RPMI + 2% inactivated Chinese-origin Rhesus monkey serum (Creative Biolabs, Shirley, NY, USA) + 2% Penicillin-Streptomycin (Sigma-Aldrich, Carlsbad, CA, USA).

For the proliferation assay, PBMCs were stained with Carboxyfluorescein succinimidyl ester (CFSE) ThermoFisher Scientific, Waltham, MA, USA) according to the manufacturer’s instructions prior to stimulation. 1x Phytohemagglutinin-L (PHA-L; ThermoFisher Scientific, Waltham, MA, USA)-stimulated cells were used as a positive control and unstimulated cells were used for background correction. To monitor cytokine production, the stimulation was performed in the presence of Golgi Stop and Golgi Plug (BD Biosciences, Franklin Lakes, NJ, USA). Unstimulated PBMCs were used as background control and PBMCs stimulated with 312.5 pg/mL Phorbol 12-myristate 13-acetate (PMA) and 125 ng/mL ionomycin (Sigma Aldrich Inc., San Diego, CA, USA) were used as positive controls.

For both assays, PBMCs were stained for CD3-PerCP-Cy5.5, CD4-BV605, CD8-BV650, CD45RA-PE-Cy7, CD28-APC-R700, CCR7-BV421, CD154-APC, CCR5-BV605 (BD Biosciences, Franklin Lakes, NJ, USA) and Fixable Aqua Dead Cell Stain (ThermoFisher Scientific, Waltham, MA, USA). To monitor the peptide responses and cytokine production, PBMCs from the 12-h assay were stained for CD69-Pe-TxRed (Beckman Coulter, Indianapolis, IN, USA), CCR5-BV786, IFN-γ-BUV395, TNF-α-FITC, (BD Biosciences, Franklin Lakes, NJ, USA) and IL-2-APC-Fire (Biolegend, San Diego, CA, USA), following intracellular permeabilization and blocking modified from previously published protocol [40]. For the 7-day assay, the CD69-Pe-TxRed (Beckman Coulter, Indianapolis, IN, USA) was stained extracellularly. Data were acquired on an LSRII Fortessa cytometer (BD System, Woburn, MA, USA) and analyzed using a FlowJo v10.7.1 (BD Biosciences, Woburn, MA, USA). Central memory T-cells were defined as CD45RA-CCR7+ T-cells, whereas effector memory T-cells were defined as CD45RA-CCR7− T-cells.

### 2.7. Statistical Analysis

Sample size was determined in coordination with the International AIDS Vaccine Initiative in light of their ongoing evaluation of VSV-HIV vaccines. Graphs and statistical analyses for antibodies and viral loads were performed using a GraphPad Prism 8 (GraphPad Software, La Jolla, CA, USA). For the T-cell response, analysis to peptide pools were first cleaned using background correction for cytokines (IFN-γ and IL-2), activation markers (CD69 and CD154) and percent-divided cells. All statistics and graphing for the T cell analysis was performed using an RStudio v2023.06.0+ (Posit, Boston, MA, USA).

## 3. Results

### 3.1. Recombinant VSV-HIV Vaccines Induce Strong Humoral Responses

**Figure 2:**
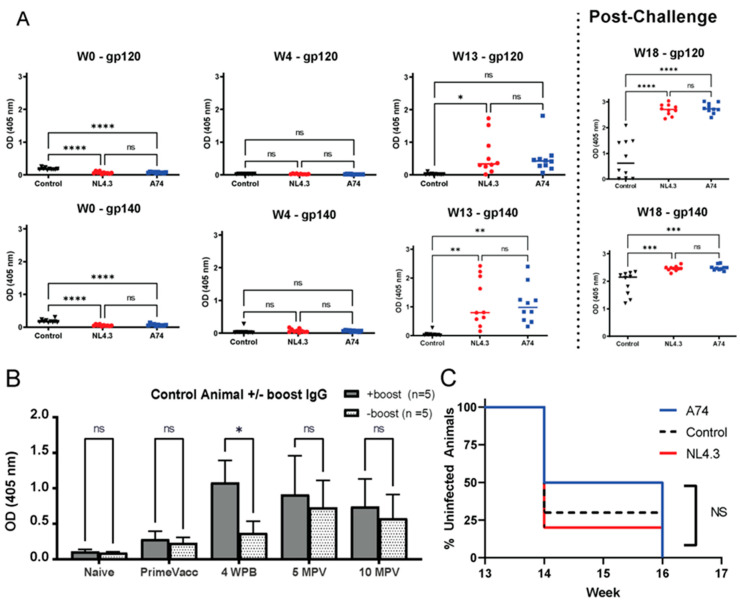
**Antibody responses and SHIV acquisition.** (**A**) Antibody responses following vaccination and boost. Binding antibodies in serum samples to HIV M consensus gp120 and gp140 proteins were analyzed by ELISA at four time points over the first 18 weeks. Comparisons were calculated using Tukey’s Multiple Comparisons Test. (**B**) Kinetics of EBOV GP-binding antibodies in serum samples of animals in the control group, with or without the boost, during the indicated time period. WPB = weeks post boost, MPV = months post prime vaccination. Comparisons were made via Sidak’s multiple comparisons. (**C**) Kaplan-Meier curves showing the acquisition rate of the vaccinated and control animals (n = 10 per group) after repeated high dose challenge with 1 × 10^4^ TCID_50_ SF162p3. Infection status was assessed one week after each challenge. OD: Optical density. * *p* < 0.05, ** *p* < 0.01, *** *p* < 0.001, **** *p* < 0.0001. ns = not significant.

Following vaccination, the magnitude of the generated humoral response against HIV Env and EBOV was measured by ELISA. Most of the VSV-Env-vaccinated animals developed binding antibodies against HIV glycoproteins gp120 and gp140 after the boost. The level of Env-specific antibodies further increased following challenge but were not correlated with viral load (Figure 2A). The control animals, which were vaccinated with VSV-B6, developed antibodies specific to EBOV GP (Figure 2B). A significant boost effect was observed in the control subgroup receiving the EBOV-GP DNA boost at week 9 compared to the mock-boosted animals. However, the control groups’ antibody responses roughly equalized at week 20 (5MPV) and remained overall stable until termination. Neutralizing activity against Env pseudovirus was assessed but could not be detected.

### 3.2. Plasma Viral Load Trends and Correlation with Rectal Viral Shedding

**Figure 3:**
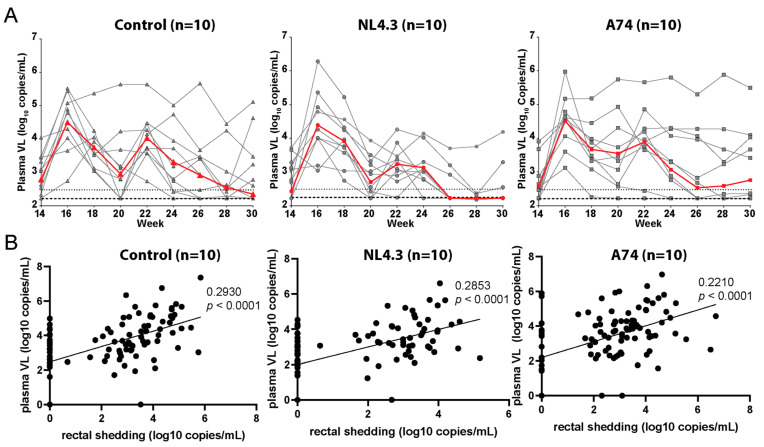
**SHIV viral loads detected in plasma and rectal shedding.** (**A**) SHIV viral loads detected in plasma every second week from week 14 to week 30. Values from individual animals are represented in grey lines. Mean viral load of the group is indicated in red. (**B**) Relationship of SHIV viral loads (VL) detected in plasma and rectal swabs during the same timespan as listed above. Individual animals are visualized as dots. RNA was isolated from plasma or rectal swabs and analyzed by RT-qPCR for the presence of Gag RNA. Correlation between values was analyzed by simple linear regression. Numerical values shown are r^2^ and *p*-values.

Thirteen weeks after the initial vaccination (4 weeks after the boost), animals were challenged intrarectally with SHIV SF162p3 (1 × 10^4^ TCID50), a heterologous clade B virus. Animals were challenged at 2-week intervals for a total of six times. Sampling was performed on the day of the first challenge (W13) and then every other week between the challenges (Figure 1C). Plasma viral loads and rectal mucosal viral shedding were determined every two weeks and followed for a total of 19 weeks for all three groups (Figure 3). Animals were considered infected when they had detectable viral loads above 300 copies/mL plasma. After the first challenge, 7/10 control animals were infected compared to 5/10 in group A74 and 8/10 in group NL4.3 (Figure 2C). After the second challenge, all animals were infected, as indicated by mean plasma titers of 2.3 × 10^5^ copies/mL in NL4.3, 1.34 × 10^5^ copies/mL in A74, and 9.35 × 10^5^ copies/mL in controls. Peak viral loads were reached 1-3 weeks after initial challenge, although secondary peaks with viral loads exceeding the primary peak were seen in the control group (n = 2), in group A74 (n = 3), and in group NL4.3 (n = 1) (Figure 3A). A decline in viral load was observed after the peak at second challenge in all groups. Two animals, one in the control group and one in group A74, displayed a pattern of consistently high viral loads throughout the study, with peak plasma titers of 4.7 × 10^5^ and 7.7 × 10^5^ copies/mL, respectively. In contrast, two animals in group A74 showed some of the lowest overall and lowest peak plasma titers and were the first two animals able to control the infection, as seen after challenges 3 (Week 18) and 5 (Week 22), respectively (Figure 3A). Most animals in group NL4.3 had a lower titer after the fourth challenge as compared to the other groups. By the end of the challenge phase (W26), only 3/10 NL4.3 animals still possessed viral loads above the limit of detection (Figure 3A).

Viral load was no longer detectable in 63% of the animals in all groups combined 5 weeks following the last challenge (week 28). However, an increase was observed again in some animals during the following two weeks, leaving a total of 53% with undetectable viral load at study termination. To follow the natural disease progression, the control animals were monitored for an additional month, at the end of which 20% of control animals had undetectable viral load.

To investigate whether the vaccines might be able to reduce the risk of transmission, rectal viral load was measured and compared to the plasma levels using simple linear regression of Log10 values (Figure 3B). In all animals, viral shedding had a significant positive correlation with the plasma levels (*p* < 0.0001). The control group (VL-Ebo) had the highest relationship between the values, with an r^2^ value of 0.293. The NL4.3 group had the next highest, with an r^2^ value of 0.285, and A74 had a value of 0.221, showing the weakest correlation of the three. The similar correlation coefficiency between the various groups suggests that the tested vaccines did not affect the likelihood of transmission. However, as the positivity cutoffs were different between the two assays, we might not have been able to detect low levels of viral shedding. Nevertheless, as we saw no vaccine effect on viral shedding, it probably does not have an important impact in this study; however, it could be optimized in future projects.

### 3.3. Cytokine Production and Activation Markers by Central and Effector Memory T-Cells

**Figure 4:**
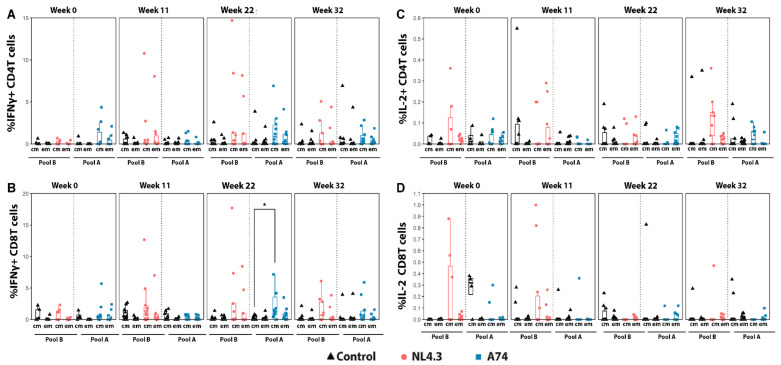
**Cytokine CD4 and CD8 T cell analyses**. (**A**,**B**) IFN-γ producing CD4 and CD8 central memory (cm) and effector memory (em) T-cells. (**C**,**D**) IL-2 producing CD4 and CD8 central and effector memory T-cells. PBMCs obtained from animals in the control group were stimulated with both pool A and pool B peptides, and the response is pictured next to the corresponding group. The y-axis indicates the percentage of stimulated cells after the subtraction of unstimulated controls. Em: Effector memory T cells. Cm: central memory T cells. * *p* < 0.05.

The T-cell response to viral antigens was evaluated at four time points: week 0 (before vaccination), week 11 (2 weeks post boost), week 22 (following five challenges) and week 32 (9 weeks after the last challenge). PBMCs were stimulated with HIV Env peptide pool A (Group A74, HIV Subtype A1) or pool B (Group NL4.3, HIV subtype B). While not significant, we observed increased IFNγ production by central memory T-cells in group NL4.3 in both CD4 and CD8 T-cells following the boost immunization but prior to infection (W11 (Figure 4A,B)). The magnitude of IFNγ production increased following SHIV challenges (W22) for both groups A74 and NL4.3, with A74 CD8 TCM cells showing a significantly higher signal than the controls. Though there were still detectable peptide responses 9 weeks following the last challenge (W32), the A74 magnitude decreased from week 22 (Figure 4B). In contrast, at week 32, NL4.3 CD8 CM cells continued to trend higher than the controls. For IL-2, much smaller magnitude changes were observed following stimulation with peptide pools (Figure 4C,D). At the initial timepoint (W0) and post boost (W11), IL-2 production by CD4 and CD8 T-cells for group NL4.3 was detected, though this distinction was lost as the weeks progressed, with a slight, non-significant boost seen in NL4.3 CD4 TCM cells at week 32.

**Figure 5:**
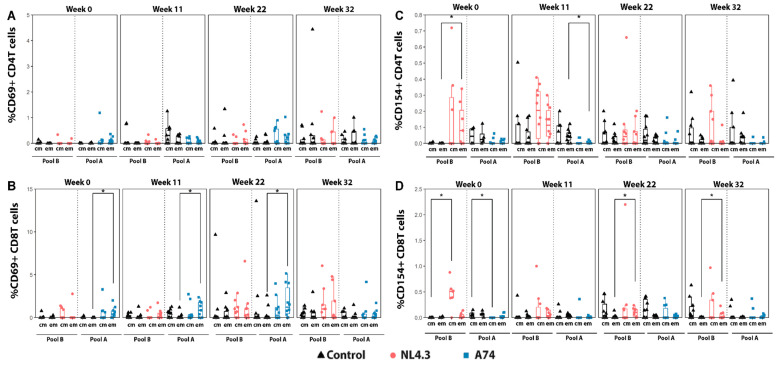
**CD69+ and CD154+ CD4 and CD8 T cell analyses**. (**A**,**B**) CD69+ CD4 and CD8 central (cm) and effector (em) T-cells. (**C**,**D**) CD154+ CD4 and CD8 central (cm) and effector (em) T-cells. PBMCs obtained from animals in the control group were stimulated with both pool A and pool B peptides and the response is pictured next to the corresponding group. The y-axis indicates the percentage of stimulated cells after the subtraction of unstimulated controls. Em: Effector memory T cells. Cm: central memory T cells. * *p* < 0.05.

The analyses of activation markers CD69 and CD154 showed conflicting trends (Figure 5). CD69 marker upregulation was almost exclusively limited to A74 CD8 TCM cells, showing a steady increase in the magnitude of response between W0 and W22, with a significant response seen at each time point (Figure 5B). However, this A74 CD8 TCM signal vanished between W22 and W32. In contrast, Group NL4.3 showed significantly higher CD154 responses than the control at timepoints W0, W22, and W32, while A74 showed a decreased response compared to the control at W0 and W11. While significantly higher at multiple points, the NL4.3 CD154-relevant cell population shifted over the study period, with W0 showing statistically higher CD4 TEM and CD8 TCM, while W22 and W32 showed a statistically higher CD154 expression in CD8 TEM cells (Figure 5C,D). This shift in CD154+ cell population potentially represents the activation of TCM to become TEM [41].

### 3.4. Proliferating Central- and Effector Memory T-Cells

To monitor systemic T-cell activation, T-cell proliferation after stimulation with Env peptide pools was monitored by flow cytometry using CFSE at four time points: week 0 (before vaccination), week 11 (2 weeks post boost), week 22 (following five challenges), and week 32 (9 weeks after the last challenge).

**Figure 6:**
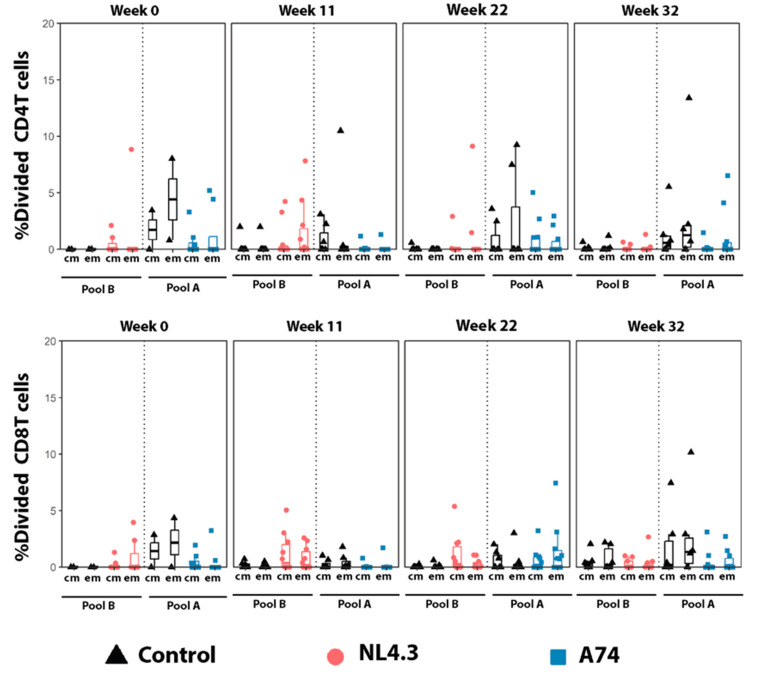
**Percentage of Proliferating T-cells.** Percentage of proliferating CD4 and CD8 central memory (cm) and effector memory (em) T cell responses. PBMCs obtained from animals in the control group were stimulated with both pool A and pool B peptides, and the response is pictured next to the corresponding group. The y-axis indicates the percentage of stimulated cells after the subtraction of unstimulated controls. Em: Effector memory T cells. Cm: central memory T cells. Data was not statistically significant.

The percentage of proliferating total, central, and effector memory cells, as well as CD45RA+ effector memory CD4+ and CD8+, were measured using CFSE. While no statistically significant differences were observed between the groups, a tendency of increased proliferation of central and effector memory T-cells 2 weeks after the boost (W11) was observed in group NL4.3 compared to the controls (Figure 6). In group A74, the percentage of dividing cells was lower than the controls for all time points. The T-cell responses were, in general, lower at week 32, 9 weeks after the last challenge. Viral load was not associated with a higher or lower systemic T-cell activation.

### 3.5. Development of an AIDS-like Syndrome in a Control-Vaccinated Animal

Even though SHIV SF162p3 is generally not considered a very “pathogenic” infection model, we observed the development of severe disease/AIDS-like syndrome in one animal in the control group. The symptom onset started 29 weeks after the first challenge and included fever, severe yellowish diarrhea, dehydration, and a ~15% weight loss. No overt neurological impairment was observed. The animal was treated with subcutaneous fluids with limited effect. Blood hematology analysis demonstrated a reduction in lymphocytes and mean corpuscular volume, as well as an increase in platelets and neutrophils. Given the lack of improvement of the condition of the animal, combined with consistently elevated viral loads and a very low CD4 count (122 CD4 cells/mm^3^), the animal was euthanized 29 weeks after initial challenge.

## 4. Discussion

In this study, we describe the in vivo evaluation of two novel VSVΔG-EBOV-HIV vaccine candidates expressing chimeric HIV Env proteins either from clade A (A74) or clade B (NL4.3) and pseudotyped with the EBOV GP. The immunological impacts of these vaccine candidates were evaluated in rhesus macaques challenged with chimeric SHIV. Animals vaccinated with either vaccine developed similar antibody and T-cell proliferation responses, but were not protected from repeated high-dose, intra-rectal challenges. All animals were infected after the first two challenges, and the peak viral loads were similar between the vaccinated and the control animals. Despite this, minor trends in immune responses were able to be identified in order to guide future experimentation.

The A74 vaccine contains the naturally occurring Env polymorphism N425K, which is hypothesized to result in a conformational change associated with bNAb production [42]. The NL4.3 Env is derived from a laboratory-adapted HIV-1 strain and has previously been shown to be able to induce strong immune responses in a rabies-based vaccine [33,43]. The EBOV GP was included to eliminate the vaccine vector’s dependency on CD4+/CCR5+ cells for replication, facilitating its in vitro production and expanding its host range, resulting in enhanced immunogenicity. These recombinant VSV-HIV vectors were therefore believed to be strong inducers of humoral immunity. A DNA platform was selected as a booster, as previous studies using viral vectors in combination with DNA have shown some protection abilities and reductions in the pre-exposure probability of infection [8,10]. DNA is known to produce both humoral and cellular responses, which would complement the humoral-driven prime without increasing an unspecific CD4+ T-cell activation-risking enhancement rather than protection [37,43]. The results showed a similar humoral response after boosting it in both groups, while the T-cell activations were slightly different, indicating that the vaccine strategy worked and that differences were related to the Env proteins and not the prime-boost regime.

The vaccine candidates were able to induce the production of binding antibodies; however, no correlation was seen between the binding antibodies and the viral load. In addition, no neutralizing activity was detected in any of the vaccinated animals. Group A74 showed a more diverse response after challenge, with outliers on both sides of the spectrum. On one end, one animal in this group had the highest viral load of any of the vaccinated animals, which was maintained throughout the study. On the other end of the spectrum, two animals in this group were the fastest of all animals to control the infection, resulting in undetectable viral loads after challenges 3 and 5, respectively. The levels of binding antibodies and cellular responses were similar between these outliers, so whether these differences were vaccine-induced or natural variations could not be determined with certainty and thus will require further evaluation. Comparing the two groups, more A74 animals remained uninfected following the first challenge (50%) compared to NL4.3 (30%). Group NL4.3 presented a more uniform response, with all animals in the group able to partially control the viral load after the initial peak. However, none of the animals were able to consistently reduce the viral load below the detection limit until after the last challenge.

One could speculate that a more balanced antibody response displaying key effector functions, rather than high non-specific responses, could provide a better environment for the development of immune protection and avoiding immune enhancement. Antibodies with neutralizing capacities were not detected in this study. However, other effector functions, which remained unexamined, may be relevant. The enhanced induction of IgG Fc-effector functions in these vaccines might be achieved by including other HIV genes, for example *gag* and *pol* in addition to the HIV Env, to broaden the effect of vaccine-induced antibodies [37,44]. That said, it was noted during our study that certain vaccinated animals had higher peak viral loads, but whether or not this is an antibody-mediated enhancement or just coincidence remains to be elucidated. Recent investigations have found that certain IgG fractions are able to increase the susceptibility of non-human primates (NHPs) to the mucosal SHIV challenge [45]. However, no clear correlation was seen between the viral load and the binding antibodies in the vaccinated groups in this study.

Looking at T-cell responses, group A74 demonstrated a CD69 response in CD8 TCM that was consistently higher than the controls, seen first at W0 and continuing through W22 well into the challenge course. CD69 acts as a non-specific signal associated with rapid immune activation following infection and has been associated with successful vaccination [46,47]. It is furthermore known to suppress the sphingosine-1-phosphate function leading to lymphocytes retention and may serve to regulate NK cell, IFN-γ, and TNF-α responses [47,48] However, as no protection was observed in the vaccinated animals, the significance of the CD69 response requires further studies.

Group NL4.3 exhibited distinct trends from A74, starting with trends of increased proliferation in all T cell populations at Week 11 prior to infection (Figure 6). This trend could be associated with infection as an increase in T cell proliferation increases the potential viral targets [49,50]. Significant CD154 activation was also seen in Group NL4.3 cells, though the relevant cell population changed over the course of the study. CD154 acts as a ligand for CD40, which impacts a myriad of downstream mechanisms, among which are antigen-presenting cell activation and maturation, T-cell priming, type 1 cytokine production, macrophage effector functions, antibody isotope switching, and germinal center formation [51,52]. The wide range of this marker’s activity makes it difficult to identify the relevant mechanisms without further study. In contrast, Group A74 exhibited neither of these trends in proliferation or CD154, instead displaying patterns similar to, if not lower than, the experimental controls, yet a larger percentage of animals (50%) endured the first round of challenge compared to the controls (20%). The implications of these and other cell-mediated forms of immunity should be investigated as in previous research [53,54].

Challenge dosage using the SF162p3 strain, study design, and timing between vaccination boosts and challenges are other points to be considered. The SF162p3 strain has been used in other studies but with low dose inoculations compared to this regimen. One 2015 study used six repeated intrarectal inoculations of 500 TCID50 SF162p3 [15]. Another 2013 study used a single intrarectal inoculation of 1000 TCID50, but this study only examined control of infection, not resistance [55]. While the dosage probably played an important role in the fast infection, the design and timing might also matter. Many studies with similar schedules to the current study (4–8 weeks post last immunization) only challenged once, while others delayed challenge until 6 to 12 months after the final boost, but challenged multiple times [5,6,56,57]. Increased time from boost to challenge might help reduce nonspecific immune reactions that favor enhancement, which would therefore be preferable for protection. In addition, most successful macaque vaccine studies used either a low-dose schedule (500–1000 TCID50) with several repeat challenges or a one-time high dose challenge (4000–5000 TCID50) to mimic some of the natural routes of transmission [6,10,12,15,56]. All of this combined would be relevant to consider for the design of future vaccine studies.

## 5. Conclusions

In the current study, two VSV-based vectors containing the Ebola glycoprotein and HIV-modified envelopes from either clade A (A74) or clade B (NL4.3) were able to produce relatively strong antibody responses in macaques. Though no protection was seen after repeated challenges with SHIV SF162p3, multiple trends in immune responses were observed. Comparisons between the vaccine groups found that Group A74 saw significantly increased CD69+ markers in T cell populations during the week 11 timepoint between the end of vaccination and the beginning of challenge, while NL4.3 saw trends of higher levels of IL-2 and CD154 and as well as higher percentages of cell proliferation. These preliminary observations match those seen in Jelinski, Kowatsch, and Lafrance et al., which observed vaccine-induced resistance independent of antibody responses and characterized by increased CD69 responses in CD8 TCM [31]. Further studies confirming these findings would be relevant for the determination of possible correlates of protection in rVSV-based vaccines.

## Data Availability

The datasets generated during and/or analysed in this study are available from the corresponding author on reasonable request.
